# END-OF-LIFE DECISION-MAKING: INFORMATION PRACTICES OF UCHINANCHU OLDER ADULTS

**DOI:** 10.1093/geroni/igae098.2028

**Published:** 2024-12-31

**Authors:** Kristina Shiroma

**Affiliations:** Louisiana State University, Baton Rouge, Louisiana, United States

## Abstract

End-of-life (EOL) decision making encompasses sensitive, yet pivotal decisions requiring nuanced information. Understanding how diverse populations understand and make EOL decisions is crucial as diverse information practices can inform policies to enhance value-centered care for a broader range of patients at end-of-life. This study aims to better understand diverse approaches to EOL decision-making from older adults’ who come from an underrepresented ethnic minority group in the United States. Reflexive thematic analysis of in-depth, semi-structured interviews with 18 Uchinanchu American older adults. Recruitment through convenience and snowball sampling for in-person and telephone interviews. Three themes were identified in Uchinanchu American older adults’ approaches to EOL decision making: Prioritizing Mitigation of Burden for Adult Children; Keeping Customs of Filial Responsibility; and Maintaining a Shoganai Mindset. Uchinanchu participants were thinking about EOL decision making both down the familial lineage as they prioritized mitigating burden for their adult children, but also up the familial lineage as they kept customs of filial responsibility for their parents. Participants’ cultural approach to shoganai (the Japanese word associated with accepting one’s fate) helped them to balance a cultural acceptance of death as a fated part of life to either make or defer from EOL decision making. This study extends our understanding of diverse approaches to end-of-life decision making for underrepresented ethnic minority older adults in the United States. Future work in EOL decision making should include hard-to-reach, underrepresented populations to provide for inclusive and representative understandings of death and dying.

